# Imperial Provision for the Sick

**Published:** 1890-06-07

**Authors:** 


### The Hospital

A Weekly Journal of

Science, Medicine, Nursing, and Philanthropy.

*For Hospitals, Asylums, Medical Practitioners, Students, Nurses, Families, Parishes, Congregations, and the Charitable Public.*

Vol. VIII.—No. 193.] [June 7, 1890.

### Imperial Provision for the Sick.

Many people are quite unaware that "Our Mother, the State" makes provision for the sick. Very few know how much provision is made, or of what quality it is. All persons who feel kindly towards the sick poor will be glad to have information upon the subject, and to have it made quite clear that either by voluntary or by state agency, the sick-poor are, on the whole, cared for in a way worthy of a Christian country. The Metropolitan Asylums Board has issued its report for the year ending March 25th, 1890. That report shows us the class of cases chiefly dealt with by State agency, the extent of ground covered, the methods employed, the measure of success attained, and the cost of the work done.

The State, by means of the Metropolitan Asylums Board, deals in London chiefly with the following classes of cases, viz., fevers, including small-pox, diphtheria, typhoid, typhus, scarlet fever and the like; lunacy cases and imbeciles; orphan and helpless children, and sick paupers generally. Cases of illness coming under any one of the above heads are taken charge of promptly and authoritatively. It is obvious that this is very important to three classes of persons: to the sufferers themselves who are thus assured of kind and skilful treatment; to their friends who are thereby relieved of responsibilities which their resources are totally inadequate to meet; and to the healthy public who are protected from the dangers of infection by the complete isolation of all infectious cases.

The Metropolitan Asylums Board, which, as we have said, is the State agency for dealing with certain classes of the sick poor in London, sets about its work in a thoroughly business-like way. Its first duty is of course to find the sick. For, as may be supposed, many of the sick poor do not know that any State provision is made for them; and even where they do know they are often very helpless in availing themselves of it. The Board on the other hand, by means of the Notification of Infectious Diseases Act, which was recently passed, is able to lay its hands at once upon all those who are attacked by infectious diseases and are, therefore, dangerous to the rest of the community. In order to show with what promptitude infectious cases can now be isolated we quote a part of Section 10 of the Notification Act: "Where a Medical Officer of Health receives, in pursuance of this Act, a certificate of a medical practitioner relating to a patient within the Metropolitan Asylums District, he shall, within twelve hours after such receipt, forward a copy thereof to the managers of that district. . . . The managers shall send weekly to the London County Council such return of the infectious diseases of which they received certificates in pursuance of this Act as the London County Council from time to time require."

The information which is thus forwarded daily, and in many cases hourly, by the various Medical Officers of Health to the Central Office of the Metropolitan Managers is invariably acted upon at once. Prompt and complete isolation is thus secured. The managers in their turn carefully tabulate all the information received from the Medical Officer of Health, and at the end of every week send to each officer a complete statement of all the infectious diseases of every kind which have been notified in the whole metropolis. Thus, both the Managers at the central office and every Medical Officer of Health in his own locality are thoroughly informed from day to day and from week to week of every fact and circumstance connected with the outbreak of any and every infectious disease. The value of this method of notification, isolation, tabulation, and periodical circulation of all facts connected with infectious diseases within the metropolitan area is incalculable.

It is not to be supposed that the Metropolitan Asylums Board renders services to the very poor only. Until lately its powers were so limited that none but actual paupers could be admitted to its admirably-managed fever hospitals and general infirmaries. That limitation was particularly hard upon all those persons who have large families, but are compelled to live in small houses. When infectious disease enters such a family there is no means of isolation because of the smallness of the house. The consequence too often is that the one case of diphtheria or scarlet fever is rapidly multiplied into five or six. Now, however, that the managers are empowered by the passing of a special Poor Law Act to admit into their hospitals persons who are not paupers, but who are suffering from fever, small-pox, diphtheria, and the like, the single case in the small but crowded house may be at once removed, and all danger to the other members of the family removed with it. The importance of this from a public point of view cannot be exaggerated. The effect of it will be to reduce the dangers from infectious diseases almost to the vanishing point. By way of rendering the powers of the Board to deal with dangerous diseases ideally complete, the same Act permits them to allow their ambulance carriages to be used for the conveyance of all kinds of persons, as well as paupers, who may be suffering from infectious disease; and not only to remove them to or from the asylums of the Board, but also to such ordinary hospitals and other places of isolation and safety, as their friends may desire. Everybody will do well to bear in mind that in future, for the modest sum of five shillings, any patient suffering from an infectious disease may be removed from any part within the metropolitan area to any other part by means of one of the comfortable and excellently appointed ambulances of the Metropolitan Asylums Board.

During the past year the Managers have shown a desire to increase the public efficiency of the various departments under their control, which is in every way commendable. They have, for example, a staff of trained nurses at each infirmary and sick asylum. But it happens periodically in London that a sudden exacerbation of some prevailing fever takes place; or less frequently, but by no means rarely, an overwhelming epidemic comes upon us. Each sick asylum or fever hospital, having no more than its ordinary staff of nurses, is thereupon thrown into dire confusion and inefficiency by the unusual strain on its resources. This ought not to be; and the managers are resolved that if possible it shall not be. They are about to organise a central staff of highly-trained superintendent nurses. Each such nurse can supervise and render efficient a dozen untrained women in an emergency. With a central staff, say of twenty such nurses, the annual cost of which will be comparatively small, the managers would feel they could send to the relief of any overstrained hospital twenty, fifty, or, if need were, a hundred efficient helpers at a moment's notice. It is to be hoped that they will organise their central nursing staff without delay.

It is obvious, even to the uninitiated, that the fever hospitals and general infirmaries of the Metropolitan Asylums Board, receiving for treatment, as they do, all the sick from the homes of the poorest classes, as well as many that are not so poor, must have within their walls an enormous amount of all kinds of material of the greatest possible value to the medical student for purposes of study. Nobody perhaps is particularly to blame, but certainly it is the fact that, up to the present time, all that material has gone absolutely to waste. Energetic and intelligent persons have protested against this inexcusable policy from time to time. To do the Board itself justice, it has not, at any rate in recent years, interposed any difficulties in the way of young men desirous of following up medical studies at its hospitals. Now, however, it has adopted a policy which is no longer one of mere passive acquiescence. Having acquired the necessary powers by Section 4 of the Poor Law Act, 1889, the General Purposes Committee are conferring with the Local Government Board, with the object of making their immense clinical resources immediately available for the purposes of medical study. To those who like to obtain full value for their money—and what sensible man does not?—the evident desire of the Metropolitan Asylums Board to put their hospitals to every possible variety of public use is eminently satisfactory.

